# Postharvest Application of *Myo*-Inositol Extends the Shelf-Life of Banana Fruit by Delaying Ethylene Biosynthesis and Improving Antioxidant Activity

**DOI:** 10.3390/foods14152638

**Published:** 2025-07-28

**Authors:** Lingyu Hu, Yi Li, Kun Zhou, Kaili Shi, Yi Niu, Feng Qu, Shenglin Zhang, Weidi He, Yuanli Wu

**Affiliations:** 1 College of Horticulture and Landscape Architecture, Southwest University, Chongqing 400716, China; hly189182@163.com (L.H.); liyi0701@email.swu.edu.cn (Y.L.); zhoukun881016@163.com (K.Z.); m19917563309@163.com (K.S.); niuy2001134@163.com (Y.N.); fengqu085578@163.com (F.Q.); konjac@163.com (S.Z.); 2 Institute of Fruit Tree Research, Guangdong Academy of Agricultural Sciences, Key Laboratory of South Subtropical Fruit Biology and Genetic Resource Utilization, Ministry of Agriculture and Rural Affairs Guangdong Provincial Key Laboratory of Science and Technology Research on Fruit Tree, Guangzhou 510640, China; 3 Guangzhou Yueguo Agricultural Technology Company Limited, Guangzhou 510640, China

**Keywords:** banana ripening, shelf-life, *myo*-inositol, ethylene biosynthesis, reactive oxygen species, cell wall loosening

## Abstract

Banana fruits are harvested and then undergo rapid ripening and senescence, sharply limiting their shelf-life and marketability. *Myo*-inositol (MI) is an important regulator in ethylene production and reactive oxygen species (ROS) accumulation; however, its involvement in the postharvest ripening process of banana remains to be determined. This study found that postharvest application of MI could efficiently delay the fruit ripening and extend the time in which the luster, color, and hardness were maintained in two cultivars with contrasting storage characteristics, storable ‘Brazil’ and unstorable ‘Fenza No. 1’, when stored at room temperature (23 °C ± 2 °C). Moreover, physiological, metabolic, and gene expression analyses indicated that MI application improved MI metabolism and postponed ethylene biosynthesis and cell wall loosening. The decrease in ethylene production was associated with a reduction in the expression of *ACS1* and *ACO1* genes. MI treatment decreased the expressions of *PL1/2*, *PG*, and *EXP1/7/8*, which may account for the delay in softening. In addition, the application of MI could alleviate ROS-mediated senescence and cell membrane damage by promoting the activities of SOD, POD, and anti-O_2_^−^ and decreasing PPO activity. This study shed light on the function of MI in regulating the postharvest ripening and senescence of bananas and provided an efficient strategy for extending shelf-life and reduce losses.

## 1. Introduction

Banana (*Musa* spp.) is one of the most popular global fruit crops because of its high nutritional and economic value [1]. As a typical climacteric fruit, postharvest banana fruit undergoes a rapid ripening process, accompanied by respiration burst, significantly increased ethylene biosynthesis, and changes in nutrients, texture, and color [2]. Postharvest ripening is essential for developing an edible quality, but over-ripening (i.e., in a sense of senescence) reduces shelf-life and marketability, resulting in serious losses [3,4]. Therefore, developing effective strategies to delay ripening and senescence is necessary for shelf-life extension and loss reduction of banana fruit.

Fruit ripening is a complex physiological and biochemical process, modulated by the interplay of environmental conditions, phytohormones, transcription factors (TFs), and epigenetic regulators, which ultimately define the fruit’s color, texture, flavor, and aroma. The hormone ethylene plays an important role in the regulation of ripening and senescence processes in climacteric fruit, including banana. However, bananas are generally harvested and transported at the mature-green stage under controlled circumstances. Thus, banana ripening is governed by ethylene biosynthesis and signaling [5,6]. Ethylene biosynthesis is controlled by two key reactions, mediated by 1-aminocyclopropane-1-carboxylic acid (ACC) synthase (ACS) and ACC oxidase (ACO), which convert S-adenosyl-L-methionine into ethylene [7,8]. Slowing ethylene production and signaling is an effective strategy for delaying the ripening and senescence of climacteric fruit [9]. It was indicated that the ripening speed of banana depends on the combination of the A and B subgenomes. The B subgenome is dominant and leads to rapid ethylene production during ripening. The ‘Fenza No. 1’ (*M.* × *pradisiaca*, Pisang Awak; ABBB group) has a larger copy of the B subgenome than the monospecific ‘Brazil’ (*M. acuminate* Cavendish cv. Baxi; AAA group) and exhibits a shorter shelf-life than ‘Brazil’ [10,11].

During fruit ripening, ethylene production also leads to an imbalance between the generation and scavenging of reactive oxygen species (ROS), resulting in physiological disorders and senescence acceleration in fruit [12,13]. ROS over-accumulation leads to lipid peroxidation and the production of toxic substances such as malondialdehyde (MDA) in fruit, resulting in membrane injury and undesirable flavors and odors [14,15]. In addition, ROS-mediated oxidation injury results in quality deterioration by accelerating the cell wall decomposition of banana fruit. Antioxidant enzymes, including catalase (CAT), superoxide dismutase (SOD), peroxidase (POD), and so forth, are vital to alleviate ROS accumulation and oxidative stress. Therefore, improving enzymatic antioxidants and inhibiting ROS over-accumulation is essential for delaying quality deterioration and extending the shelf-life of fruit [16,17].

Excessive softening is undesirable, because it accelerates pathogen infection and increases losses [18]. Fruit softening is a complex process, mainly due to cell wall decomposition, particularly depolymerizing hemicellulose and pectin [19,20]. Pectin is a major part of the composition of the primary cell wall and plays an important role in keeping the mechanical intensity and stability of the physical structure [21]. Previous studies suggested the involvement of many enzymes in the process of fruit softening. Among these, pectin methylesterase (PME), polygalacturonase (PG), and pectate lyase (PL) are responsible for the disassembly of pectin [22,23]. Expansin proteins (EXPs) and xyloglucan endotransglucosylase-hydrolase participate in disassembling or modifying hemicellulose [18,24].

*Myo*-inositol (MI) is a widely found polyol that acts as a precursor for the biosynthesis of multiple compounds, such as inositol polyphosphates, raffinose, phosphatidylinositol phosphate, pinitol, and ononitol [25,26]. In higher plants, MI metabolism plays an important role in stress tolerance, pathogen resistance, cell wall formation, programmed cell death, and hormonal regulation [27,28]. Exogenous application of MI could mitigate salt-induced injury by inhibiting ROS accumulation and maintaining osmotic balance in *Malus hupehensis* [29]. In *Arabidopsis* seedlings, exogenous MI could modulate the expression of genes related to stress tolerance, redox regulation, and cell wall deposition [30]. Furthermore, the role of MI in scavenging ROS was characterized using a transgenic approach in apple [31]. In particular, our previous research showed that inhibiting MI biosynthesis in transgenic apple lines caused ROS over-accumulation and increased ethylene production, which could be mitigated by MI complementation [32]. Considering the positive potential of MI in inhibiting ROS accumulation and ethylene production, we argued that MI may exhibit a strong potential in controlling fruit ripening and senescence, especially for the climacteric fruit banana. However, the role of MI in the shelf-life of banana fruit has received less attention. This study analyzed the effects of MI application on shelf-life, fruit appearance, respiration rate, ethylene production, and ROS accumulation during the ripening of postharvest banana fruit using storable ‘Brazil’ and unstorable ‘Fenza No. 1’. Results indicated that the postharvest application of MI improved endogenous MI metabolism and delayed the ripening and senescence of fruit in both cultivars. Furthermore, the positive role of MI application was attributed to the inhibition of ethylene biosynthesis, cell wall loosening, and ROS-mediated senescence. Our results provide a new, efficient, and pollution-free strategy for reducing postharvest loss of banana, which may benefit its high-quality development in the future.

## 2. Materials and Methods

### 2.1. Plant Materials and Chemicals

Fruits of two banana cultivars, ‘Brazil’ and ‘Fenza No. 1’, were harvested from a local farm in Guangzhou (Guangdong Province, China; 23°13′ N, 113°27′ E) in their commercial maturity (ripe green) stage. Fruits with a uniform shape, size, and color and without visible disease or damage were chosen and soaked in 2 g L^−1^ sodium hypochlorite solution for 10 min to eliminate potential microbes. Then, the fruits were washed with sterile deionized water and then air-dried at room temperature (23 °C ± 2 °C) for treatment and storage. The source for MI was Yuanye Bio-Technology Co., Ltd. (Shanghai, China).

### 2.2. Screening for the Optimum Concentration of MI and Storage Treatment

‘Brazil’ fruits were selected randomly and immersed in a solution of 0 µmol L^−1^, 50 µmol L^−1^, 100 µmol L^−1^, 200 µmol L^−1^, and 300 µmol L^−1^ MI for 2 h. Each treatment included 20 fruits and was maintained until the fruit began to decay (i.e., 18 days). Based on the screening results of MI concentrations, the fruits of ‘Brazil’ and ‘Fenza No. 1’ were separated into 2 groups randomly. The fruits in one group were dipped in a solution of 200 µmol L^−1^ MI for 2 h, whereas those in the other group were treated with sterile dH_2_O as the control. Each group included 100 fruits, and the length of storage was 14 and 7 days for ‘Brazil’ and ‘Fenza No. 1’, respectively. This storage time was set according to the fruits fully ripening at room temperature. All air-dried fruits were stored at room temperature and 80–90% relative humidity.

### 2.3. Measurement of the Pulp Firmness and Peel Color

Pulp firmness was determined at three equidistant points around the middle position of the banana fruits using the Model GY-3 penetrometer apparatus (Zhejiang Scientific Instruments, Hangzhou, China), and the results were recorded in Newton (N). The diameter of the round probe was 8.0 mm, which was used to measure the penetration force on the slices with a descent speed of 10 mm s^−1^ and a maximum insertion distance of 10 mm. The average of the three measures was expressed as the value of firmness for one banana pulp.

Peel color was detected by the Minolta Chroma Meter CR-400 (Minolta Camera Co. Ltd., Osaka, Japan). The peel color was expressed using the CIE L*, a*, and b* values.

### 2.4. Determination of Respiration Rate and Ethylene Production

The respiration rate was measured using a LI-6262 CO_2_/H_2_O analyzer (Lincoln, NE, USA). Fruits from each treatment were weighed and placed in an airtight container connected to the LI-6262 CO_2_/H_2_O analyzer. For each analysis, data were recorded every 30 s by the instrument and used to calculate the respiration rate.

Ethylene production was measured using the method proposed by Zhu et al. [33], with minor revisions. Briefly, fruits were placed in a 2.6 L airtight container, and the gas sample was collected from the container by a syringe. The ethylene content was analyzed with the GC-14A gas chromatograph (Shimadzu, Kyoto, Japan), equipped with a flame ionization detector and an activated alumina column (200 cm × 0.3 cm). The temperature settings for the injector, detector, and oven were 150 °C, 70 °C, and 70 °C, respectively, and the carrier gas flow rates were set at 300 mL, 30 mL, and 30 mL min^−1^ for air, H_2_, and N_2_, respectively.

### 2.5. Measurement of Antioxidant Enzyme Activities and Oxidative Damage

Anti-O_2_^−^, POD, and SOD activities were detected using corresponding assay kits according to the operation instructions (Nanjing Jiancheng Bioengineering Institute, Nanjing, China). Hydrogen peroxide (H_2_O_2_) and MDA contents were detected using an assay kit following the manufacturer’s instructions (Nanjing Jiancheng Bioengineering Institute, Nanjing, China). The materials used in this experiment were fresh samples.

### 2.6. Measurement of Soluble Sugar Alcohol Content

Soluble sugar alcohols were analyzed by Metware Biotechnology Technology Co., Ltd. (Wuhan, China). Briefly, the frozen samples were dried under conditions of freezing temperature and vacuum and then pulverized in liquid nitrogen. Further, 20 mg of sample was mixed in 0.5 mL of extracting solution [water: isopropanol: methanol = 2:3:3 (v:v:v)] at 4 °C for 30 min by ultrasonication, followed by centrifugation at 12,000× *g* for 3 min. After that, 50 μL of supernatant liquid was transferred to a centrifuge tube, and 20 μL of internal standard (1000 μg mL^−1^) was added before drying under N_2_ gas. The samples were then derivatized with 100 μL of pyridine methoxyamine hydrochloride solution (15 mg mL^−1^) at 37 °C for 2 h, followed by 100 μL of BSTFA at 37 °C for 30 min. A 20 μL derivative was attenuated to 1 mL using n-hexane and filtered through a syringe filter (0.22 μm) before GC-MS measurement. The sugar levels were calculated using the Agilent 7890B-7000D platform (Agilent Technology, Palo Alto, CA, USA).

### 2.7. Reverse Transcription Quantitative Polymerase Chain Reaction (RT-qPCR) Analysis

Total RNA was isolated using a Plant RNA Isolation Kit (Nanjing Vazyme Biotech Co., Ltd., Nanjing, China). First-strand cDNA synthesis was performed with a PrimeScript RT Reagent Kit, which includes a gDNA Eraser (TaKaRa, Tokyo, Japan). The RT-qPCR analysis was conducted by employing a StepOne Plus Real-time PCR Detection system (Applied Biosystems, Foster City, CA, USA) and SYBR Premix Ex Taq II (TaKaRa) according to the method outlined previously by Zhou et al. [34]. The level of relative expression for each gene was calculated using the 2^−ΔΔCT^ method, and banana actin was used as the reference gene. The primer sequences for RT-qPCR are shown in Table A1.

### 2.8. Statistical Analysis

All experimental data were expressed as the mean ± standard deviation (SD) of three biological replicates. SPSS software (version 17.0) and one-way analysis of variance were used for statistical analysis.

## 3. Results

### 3.1. Screening of Exogenous MI Concentrations

**Figure 1:**
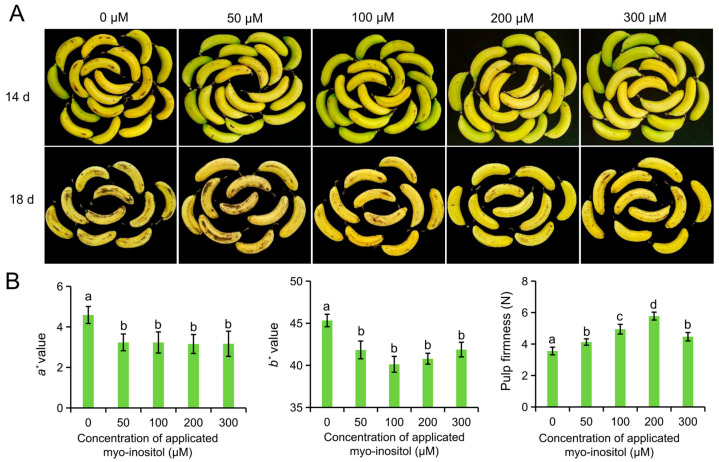
Impact of *myo*-inositol concentration on shelf-life in ‘Brazil’ fruit. (**A**) Pictures of fruit treated with different concentrations of *myo*-inositol at 14 and 18 days of postharvest storage. (**B**) Color values a*, b*, and pulp firmness on day 18. Data are means ± SD (*n* = biological replicates, each replication included 6–7 fruits). Values with different letters are significantly different (*p* < 0.05).

We initially focused on the optimal MI concentration for subsequent experimentation. Different concentrations of MI (0 µmol L^−1^, 50 µmol L^−1^, 100 µmol L^−1^, 200 µmol L^−1^, and 300 µmol L^−1^) were applied to harvested ‘Brazil’ bananas, and their storage performances were observed. The color and pulp firmness of the MI-treated groups were better than those in the control group (0 µmol L^−1^) during storage. Obvious brown spots appeared on day 14 and aggravated on day 18 in the control group, but a lower degree of brown spots was shown in the MI-treated groups. Therein, the lowest browning was observed in the fruit treated with 200 µmol L^−1^ MI (Figure 1A). The peel color of the fruits changed from green to yellow, and the values of a* and b* became higher during postharvest ripening. The a* and b* values after applying 50 µmol L^−1^–300 µmol L^−1^ MI were lower than those in the fruit of the control group. However, no significance was found between the MI-treated groups on day 18 (Figure 1B). Also, pulp firmness is a representative indicator of fruit storage quality. It reflects the level of fruit ripening and senescence. The firmness values were higher in the MI-treated groups relative to the control group, and the highest value was detected in the group treated with 200 µmol L^−1^ MI (Figure 1B). These parameters indicated that MI application could extend the shelf-life of banana fruit. Moreover, we chose 200 µmol L^−1^ MI as the concentration for further investigations.

### 3.2. Effects of MI Application on Postharvest Fruit Ripening and Senescence

**Figure 2:**
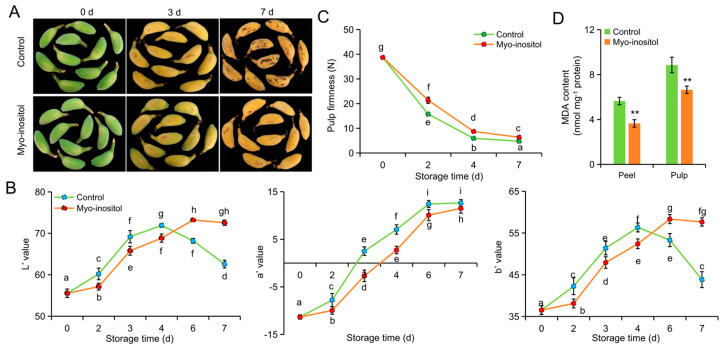
The effects of *myo*-inositol application on the postharvest storage of ‘Fenza No. 1’. (**A**) Representative pictures; (**B**) color values L*, a*, and b*; and (**C**) pulp firmness of fruit treated with *myo*-inositol during the postharvest storage. (**D**) MDA content on day 7 of storage. Data are means ± SD (*n* = 3 biological replicates, each replication included ten fruits). Values with different letters are significantly different (*p* < 0.05). In comparison with the control group, ** *p* < 0.01. Control, 0 µmol L^−1^ *myo*-inositol application; Myo-inositol, 200 µmol L^−1^ *myo*-inositol application.

The unstorable cultivar ‘Fenza No. 1’ was selected for comparison with the storable cultivar ‘Brazil’ to analyze the effect of MI application in postharvest ripening and senescence of banana fruit. As shown in Figure 2A, the peel became brown on day 7 in the control group of ‘Fenza No. 1’ but not in the MI-treated group. The L* and b* values increased and then decreased, whereas the a* value consistently increased during storage (Figure 2B). The L* and b* values were lower in the MI-treated group before day 4 but then higher than those in the control group. A lower a* value was consistently shown in the MI-treated group compared with the control group (Figure 2B). In addition, the pulp firmness gradually decreased in both groups during storage. However, the firmness of the control group was consistently lower than that of the MI-treated group (Figure 2C). MDA is the most important product of membrane lipid peroxidation and is considered a parameter in plant senescence physiology [35]. The MDA contents of both peel and pulp were significantly lower on day 7 in the MI-treated group relative to the control group (Figure 2D).

**Figure 3:**
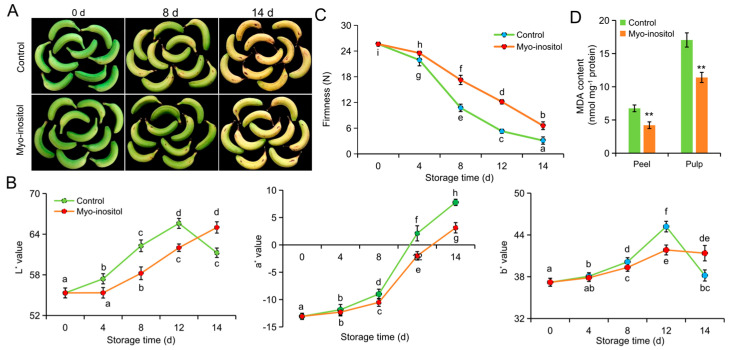
The effects of *myo*-inositol application on the postharvest storage of ‘Brazil’. (**A**) Representative pictures; (**B**) color values L*, a*, and b*; and (**C**) pulp firmness of fruit treated with *myo*-inositol during the postharvest storage. (**D**) MDA content on day 14 of storage. Data are means ± SD (*n* = 3 biological replicates, each replication included ten fruits). Values with different letters are significantly different (*p* < 0.05). In comparison with the control group, ** *p* < 0.01. Control, 0 µmol L^−1^ *myo*-inositol application; Myo-inositol, 200 µmol L^−1^ *myo*-inositol application.

A similar effect of MI in postharvest fruit ripening and senescence was observed in ‘Brazil’ (Figure 3A). The browning process was delayed in postharvest ‘Brazil’ compared with ‘Fenza No. 1’, reflecting postponed dynamics in the values of a*, b*, and L* (Figure 3B). Moreover, the slowing of MI on ripening and senescence was evident by these parameters during storage (Figure 3B). Meanwhile, fruits treated with MI still harbored a higher flesh firmness compared with the control fruits (Figure 3C). Lower MDA contents were observed in both peel and pulp of fruit treated with MI on day 14 (Figure 3D). However, comparisons of the above-mentioned values between the two cultivars indicated a higher effect of MI application on delaying fruit ripening and senescence in storable ‘Brazil’ (Figure 2 and Figure 3). Even so, we argued that MI application could efficiently delay postharvest ripening and senescence and extend the shelf-life of banana.

### 3.3. Effects of MI Application on Respiration Rate and Ethylene Production

**Figure 4:**
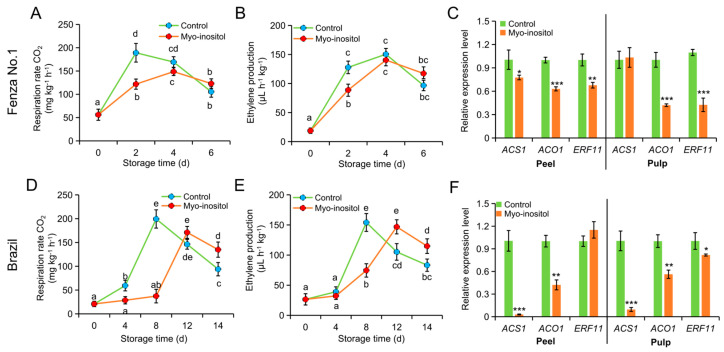
The effects of *myo*-inositol application on ethylene production during postharvest storage. Changes in (**A**,**D**) respiration rate, (**B**,**E**) ethylene level, and (**C**,**F**) expression of ethylene-related genes in (**A**–**C**) ‘Fenza No. 1’ on day 2 and (**D**–**F**) ‘Brazil’ on day 8 of storage. Data are means ± SD (*n* = 3 biological replicates, each replication included ten fruits). Values with different letters are significantly different (*p* < 0.05). In comparison with the control group, *** *p* < 0.001; ** *p* < 0.01; * *p* < 0.05. Control, 0 µmol L^−1^ *myo*-inositol application; Myo-inositol, 200 µmol L^−1^ *myo*-inositol application.

Consistent with storability, the peaks of respiration and ethylene production were advanced in ‘Fenza No. 1’ compared with ‘Brazil’ under normal conditions. Moreover, postharvest application of MI significantly delayed the progress of fruit respiration and ethylene production in both ‘Fenza No. 1’ and ‘Brazil’ (Figure 4). The respiration rate of ‘Fenza No. 1’ fruit was suppressed by MI treatment and recovered on day 4. However, the recovered rate of respiration was lower than the peak value in the control group on day 2 (Figure 4A). The ethylene production was also delayed by MI application, whereas no significance was found in the peak value (Figure 4B). The peak values of respiration rate and ethylene production appeared in ‘Brazil’ on day 8 in the control group but were postponed to day 12 by MI application. Nevertheless, MI application did not change the peak value of these two indices. In addition, these two values for fruit treated with MI were higher than those of the control group on day 14 but were comparable on day 12 (Figure 4D or Figure 4E).

Considering, however, that the physiological responses of MI application began on day 2 and 8 in ‘Fenza No. 1’ and ‘Brazil’, respectively (Figure 2, Figure 3 and Figure 4), the changes in gene expression would occur in the early stage of the physiological response; we therefore chose samples from these time points for expression analysis. Two key ethylene biosynthesis-related genes (i.e., *ACS1* and *ACO1*) and *ethylene response factor 11* (*ERF11*) were included. In ‘Fenza No. 1’, RT-qPCR analysis indicated that the transcription of *ACO1* and *ERF11* was repressed by MI application in both peel and pulp. However, the transcription of *ACS1* was only repressed in the peel and did not change in the pulp (Figure 4C). In ‘Brazil’, MI application significantly inhibited the transcription of *ACS1* and *ACO1* in both peel and pulp but only caused *ERF11* downregulation in the pulp (Figure 4F). Although gene expression differences existed, all results from the two cultivars still suggested that the application of MI postponed the postharvest ripening in banana fruit by delaying ethylene biosynthesis.

### 3.4. Effects of MI Application on the Accumulation of Soluble Sugars and Cell Wall Modification

Considering the direct contact with the storage atmosphere, we analyzed the profiles of soluble sugars in the peel of ‘Fenza No. 1’ on day 2 and ‘Brazil’ on day 8. In total, 20 classes of sugar were detected in all samples, of which 17 exhibited changes in their levels. MI application significantly decreased the accumulation of total sugar and nine sugars (xylose, sorbitol, ribono-1,4-lactone, glucose, galactose, fucose, fructose, arabinose, and arabinitol) in both cultivars. In contrast, in response to MI application, the levels of maltose and mannose were only decreased in ‘Fengza No. 1’, and trehalose, sucrose, glucuronic acid, and galacturonic acid were only decreased in ‘Brazil’. These results coincided with the delayed postharvest ripening mediated by MI application, because starch is hydrolyzed and soluble sugars accumulate during fruit ripening. Even so, the levels of MI and its downstream product raffinose significantly increased in both cultivars, indicating that MI application could improve endogenous MI accumulation and its metabolism (Table 1).

**Figure 5:**
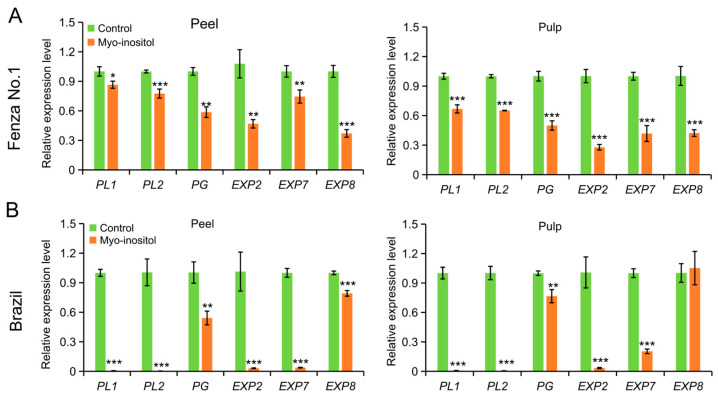
The effects of *myo*-inositol application on the expression of genes involved in cell wall degradation during postharvest storage. Expression changes in peel and pulp of (**A**) ‘Fenza No. 1’ on day 2 and (**B**) ‘Brazil’ on day 8 of postharvest storage. Data are means ± SD (*n* = 3 biological replicates, each replication included ten fruits). In comparison with the control group, *** *p* < 0.001; ** *p* < 0.01; * *p* < 0.05. Control, 0 µmol L^−1^ *myo*-inositol application; Myo-inositol, 200 µmol L^−1^ *myo*-inositol application.

Galacturonic acid is the main component of pectin [21]. The decrease in galacturonic acid levels in the MI-treated samples led us to examine whether the improved MI metabolism could modulate the expression level of genes involved in cell wall breakdown. The expression levels of two endo-pectate lyase genes, *PL1* and *PL2*; one endo-polygalacturonase gene, *PG*; and three expansion genes, *EXP2*, *EXP7*, and *EXP8*, were examined in both peel and pulp of ‘Fenza No. 1’ on day 2 and ‘Brazil’ on day 8. Except for the unchanged *EXP8* level in the pulp of ‘Brazil’, the application of MI downregulated the transcription of these genes in all remaining samples (Figure 5). Considering the expression changes in genes involved in cell wall modifications and ethylene biosynthesis, it was likely that MI application delayed the postharvest ripening of banana fruit via targeting ethylene biosynthesis and its mediated cell wall disassembly.

### 3.5. Effects of MI Application on H_2_O_2_ Accumulation and Activity of Antioxidant Enzymes

**Figure 6:**
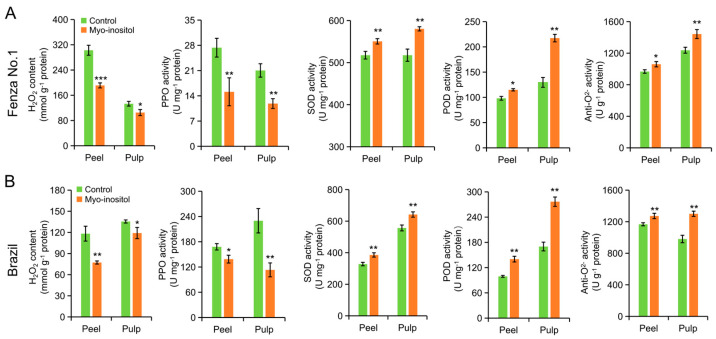
The effects of *myo*-inositol application on H_2_O_2_ level and activity of antioxidant enzymes during postharvest storage. Changes in H_2_O_2_ level and activities of PPO, SOD, POD, and anti-O_2_^−^ in peel and pulp of (**A**) ‘Fenza No. 1’ on day 7 and (**B**) ‘Brazil’ on day 14. Data are means ± SD (*n* = 3 biological replicates, each replication included ten fruits). In comparison with the control group, *** *p* < 0.001; ** *p* < 0.01; * *p* < 0.05. Control, 0 µmol L^−1^ *myo*-inositol application; Myo-inositol, 200 µmol L^−1^ *myo*-inositol application.

When fruits age, ethylene-mediated ROS accumulation in cells has been reported to cause oxidative stresses and, in turn, accelerate senescence, an inevitable process that is followed closely by ripening [12,13]. The application of MI delaying the fruit senescence was determined by analyzing the H_2_O_2_ level and the enzymatic antioxidants in the peel and pulp. As shown in Figure 6, similar results were observed in both ‘Fenza No. 1’ (on day 7) and ‘Brazil’ (on day 14). The H_2_O_2_ content and polyphenol oxidase (PPO) activity were significantly inhibited in both the peel and pulp of the MI-treated group, which fitted with their peel appearance and MDA content (Figure 2 and Figure 3). However, the higher level of ethylene production on day 12 and 14 in MI-treated ‘Brazil’ fruits relative to the control group suggested that an ethylene-independent regulation on ROS accumulation by the action of MI application also existed (Figure 4). MI has been reported to improve the activity of antioxidant enzymes and, in turn, maintain ROS balance, especially under stress conditions [32]. As expected, the activities of the detected antioxidant enzymes, that is, SOD, POD, and anti-O_2_^−^, in both peel and pulp of two cultivars at the end of storage were significantly improved by applying MI (Figure 6). These results indicated that MI application improved the activity of antioxidant enzymes and delayed ROS-mediated fruit senescence in bananas.

## 4. Discussion

Banana is a climacteric and perishable fruit. Delaying fruit ripening and senescence and prolonging shelf-life are essential for the banana industry. MI and its derivatives are ubiquitous metabolites and vital in plants’ development and stress resistance [26,32]. Our previous study found that MI was vital for fine-tuning the ROS balance and ethylene production in apples [32], indicating an important role of MI in delaying damage caused during storage and prolonging shelf-life. The present study revealed that the postharvest application of MI could efficiently extend the shelf-life of fruit by delaying ethylene biosynthesis and enhancing enzymatic antioxidants. Consistent with our study, MI application was also reported to delay the post-ripening of Chinese plum fruit [36].

The process of banana ripening can be divided into both the changes in the pulp and in the peel, and the ripening process, including softening, occurs outward from the inside of the banana, with pulp ripening preceding peel yellowing. However, the peel browning would ultimately result in disease susceptibility and decay [37]. Ethylene is a vital hormone for regulating climacteric fruit ripening, including bananas. Ethylene production triggers banana ripening to promote fruit quality formation; however, it also accelerates fruit softening and senescence to decrease shelf-life [38,39,40]. ACS and ACO are the key rate-limiting enzymes of ethylene biosynthesis and are encoded by a multi-gene family in plants [11,41]. Earlier studies found that the expression of *ACS1* and *ACO1* increases significantly at banana ripening onset, consistent with ethylene production [42,43]. Consistent with the repression in the expression of *ACS1* and *ACO1*, MI application delayed the ethylene production and ripening of two cultivars during postharvest storage of fruit. This observation was consistent with the fact that downregulation of MI biosynthesis stimulated the ethylene burst in apples [32], because MI application improved the endogenous MI accumulation and metabolism (Table 1). Moreover, our results showed that the amplitude of MI-mediated *ACS1* downregulation was smaller in ‘Fengza No. 1’ relative to ‘Brazil’, but the change in *ACO1* expression was comparable in both cultivars. This was consistent with the observation that less MI-mediated decreases in ethylene production and pulp firmness were observed for ‘Fengza No. 1’ than ‘Brazil’ (Figure 3, Figure 4 and Figure 5). The difference in the repression of ethylene production may be a key to the performance of postharvest storage in response to MI application in two cultivars. However, it was not clear why the response of *ACS1* expression to MI application was different in both cultivars. This phenomenon may be at least partially attributed to their big difference in genetic background, i.e., ‘Fenza No. 1’ (ABBB) vs. ‘Brazil’ (AAA). After all, it was indicated that the subgenomes A and B, as well as their combination, play a different role in regulating ethylene production during ripening [11].

Regulation of the ACS and ACO genes throughout the ripening of fruit, such as apples and bananas, is believed to be controlled by ERFs and other TFs [44,45]. ERFs regulate the ethylene signaling transduction to regulate multiple aspects of fruit ripening, including aroma, color, texture, and flavor [46,47]. *ERF11* is downregulated in banana fruit by ethylene treatment or during ripening process [43]. The unchanged expression in peel and minor downregulation in pulp in MI-treated ‘Brazil’ fruit was consistent with their lower ethylene production when compared with that in ‘Fengza No. 1’ (Figure 4). Furthermore, *ERF11* orthologs have been reported to act as potential repressors that bind to the promoters of *ACS1* to suppress their transcription by interacting with histone deacetylase *HDA1* [43]. This was also supported by the out-of-step expression pattern of *ACS1* and *ERF11* in the MI-treated fruits of ‘Fengza No. 1’ and ‘Brazil’ (Figure 4). This result was likely attributed to a negative feedback regulation of ethylene biosynthesis in banana fruit [48].

In addition, the downregulation of *ERF11* may be involved in the direct or indirect repression of cell wall modification. *ERF11* participated in the aril cracking of *Torreya grandis* by upregulating the transcription of *EXPs* [49]. When compared with ‘Fengza No. 1’, the unchanged (peel) or minor downregulation (pulp) of *ERF11* matched well with the higher extent of downregulation of *EXP2* and *EXP7* in ‘Brazil’ (Figure 5). Fruit softening is regulated by cell wall modification, in which EXPs promote cell wall relaxation and PLs and PGs are involved in pectin degradation [50]. Similarly, the induced ERF102 targeted and stimulated the expression of cell wall degradation genes during postharvest ripening in banana fruit [51]. Ethylene governs the transcription level of genes related to cell wall loosening and degradation [52]. Our findings showed that MI application downregulated the transcription level of genes related to cell wall loosening and degradation, that is, *PL1*, *PL2*, *PG*, *EXP2*, and *EXP7*, in banana fruit. Moreover, when compared with ‘Fengza No. 1’, the higher extent of downregulation of *PL1*, *PL2*, *EXP2*, and *EXP7* also fit with the lower ethylene production in ‘Brazil’ (Figure 4 and Figure 5). Given the leading role of ethylene burst in ripening [38], MI application may inhibit ethylene biosynthesis and, in turn, delay ripening, including cell wall degradation and loosening. Nevertheless, we did not exclude the possibility of the direct regulation of MI on cell wall modifications, because MI accumulation was closely related with pectin metabolism. The decreased MI biosynthesis induced the accumulation of soluble pectin in *MIPS1/2* and *UGT88F1*-silencing apple lines [32,53]. MI treatment was also reported to regulate cell wall metabolism and delay the postharvest softening of Chinese plum fruit [36].

Besides the critical role of ethylene in banana ripening, ROS production is natural and inevitable and, in turn, accelerates the transition from ripening to fruit senescence [12,16,38]. Excessive ROS cause oxidative damage that is harmful to specific mitochondrial proteins and, in turn, stimulates oxidative damage and quality deterioration, thereby causing fruit senescence. However, senescence will result in disease susceptibility and decay due to the weakening of cell wall strength and oxidative damage [12]. Ethylene appears to be a key factor in ROS-induced cell death regulation [54]. Consistent with our results, Ge et al. reported that hydrogen sulfide alleviated ethylene-caused postharvest senescence of banana by reducing oxidative damage [55]. However, our results observed an unchanged peak value of ethylene production during storage and a higher level of ethylene production in MI-treated fruit relative to the control group at the end of storage (Figure 4). This suggested an ethylene-independent regulation of ROS accumulation by MI application during fruit senescence. An array of non-enzymatic and enzymatic antioxidants was effectively activated to inhibit ROS over-accumulation and reduce oxidative injury in plants. Non-enzymatic systems such as fructose, glucose, sucrose, and sorbitol are protective in terms of the ROS detoxification and stabilization of membrane integrity and enzymes [32,56,57]. However, a lower level of soluble sugars, including xylose, sorbitol, ribono-1,4-lactone, glucose, galactose, fucose, fructose, arabinose, and arabinitol, was found in MI-treated peel relative to the control group (Table 1). This result indicated that enzymatic antioxidants may be the major contributors to scavenging ROS during fruit senescence in response to postharvest MI application. Moreover, our previous studies indicated that MI is vital to improve the enzymatic antioxidant system to scavenge ROS under abiotic stresses [29,31,32]. PPO exists in plants in response to various stresses, which catalyzes the conversion of phenolic compounds into quinone. Moreover, the natural redox of quinone promotes ROS production [58]. Apart from the alleviation of ethylene-mediated ROS production, MI application did improve the activities of anti-O_2_^−^, SOD, and POD and inhibited PPO activity in banana fruit, thus delaying ROS-mediated senescence and fruit deterioration.

## 5. Conclusions

Our research indicated that MI application could slow the process of postharvest banana fruit ripening and senescence and extend shelf-life by delaying ethylene production and cell wall loosening, as well as improving antioxidant activity. Due to its wide distribution and environmental friendliness, MI may be a new, efficient, and pollution-free strategy for the postharvest storage of banana fruit.

## Figures and Tables

**Table 1:** Level changes in soluble sugars in the peel of ‘Fengza No. 1’ and ‘Brazil’ treated with *myo*-inositol after 2 and 8 days of postharvest storage, respectively. Data are means ± SD (*n* = 3 biological replicates, each replication included ten fruits). In comparison with the control group, *** *p* < 0.001; ** *p* < 0.01; * *p* < 0.05. Control, 0 µmol L^−1^ *myo*-inositol application; Myo-inositol, 200 μmol L^−1^ *myo*-inositol application. FC, fold change. Compounds noted with (a) indicate a unit of mg g^−1^, and the unit of remaining compounds is μg g^−1^.

Compounds	‘Fengza No. 1’ (Day 2)			‘Brazil’ (Day 8)		
	Control	Myo-Inositol	FC	Control	Myo-Inositol	FC
Maltose	265.22 ± 6.34	196.91 ± 59.61	0.74	44.32 ± 8.69	23.1 ± 0.56	0.52 *
Trehalose	22.92 ± 0.12	13.56 ± 0.17	0.59 ***	8.73 ± 0.35	8.57 ± 0.26	0.98
Sucrose	58.56 ± 0.76	41.69 ± 2.47	0.71 ***	37.44 ± 1.37	35.77 ± 0.07	0.96
Mannose ^(a)^	36.90 ± 1.55	32.88 ± 3.14	0.89	29.48 ± 1.88	24.32 ± 0.50	0.83 *
Glucuronic acid	12.39 ± 0.04	9.56 ± 0.40	0.77 ***	5.01 ± 0.55	5.33 ± 0.12	1.07
Galacturonic acid	3.64 ± 0.01	2.95 ± 0.11	0.81 ***	2.54 ± 0.07	2.49 ± 0.03	0.98
Xylose	8.20 ± 0.08	3.65 ± 0.38	0.44 ***	5.23 ± 0.17	3.75 ± 0.08	0.71 ***
Sorbitol	95.59 ± 1.87	75.09 ± 6.82	0.79 **	22.16 ± 0.56	14.81 ± 0.02	0.67 ***
Ribono-1,4-lactone	1.35 ± 0.13	0.57 ± 0.28	0.42 **	0.99 ± 0.03	0.63 ± 0.17	0.64 *
Glucose ^(a)^	20.54 ± 0.49	11.62 ± 0.86	0.57 ***	8.27 ± 0.75	5.37 ± 0.09	0.65 **
Galactose	433.55 ± 11.26	196.22 ± 34.41	0.45 ***	82.74 ± 0.84	59.63 ± 0.63	0.72 **
Fucose	47.96 ± 0.84	37.52 ± 1.10	0.78 ***	105.86 ± 7.06	91.66 ± 2.59	0.87 *
Fructose ^(a)^	15.25 ± 0.42	8.50 ± 0.59	0.56 ***	6.40 ± 0.38	4.17 ± 0.08	0.65 **
Arabinose	9.04 ± 0.28	8.12 ± 0.16	0.90 **	9.78 ± 0.63	8.61 ± 0.30	0.88 *
Arabinitol	3.30 ± 0.75	1.52 ± 0.25	0.46 *	1.67 ± 0.34	0.50 ± 0.26	0.30 **
2-Acetamido-2-deoxy-D-glucopyranose	8.65 ± 0.08	8.36 ± 0.08	0.97	6.58 ± 0.16	6.00 ± 0.48	0.91
Rhamnose	4.66 ± 0.01	5.11 ± 0.15	1.10	7.68 ± 1.45	7.64 ± 0.18	1.00
Levoglucosan	23.52 ± 0.29	25.49 ± 1.46	1.08	16.43 ± 0.13	15.87 ± 1.92	0.97
*Myo*-inositol	28.70 ± 0.27	38.81 ± 2.17	1.35 **	49.18 ± 7.58	70.81 ± 0.99	1.44 **
Raffinose	55.88 ± 0.40	69.76 ± 5.84	1.25 *	19.73 ± 0.82	23.05 ± 1.62	1.17 *
Total sugars ^(a)^	95.41 ± 1.65	62.54 ± 4.01	0.66 ***	52.53 ± 2.50	45.68 ± 0.15	0.87 **

## Data Availability

The original contributions presented in the study are included in the article. Further inquiries can be directed to the corresponding authors.

## Appendix A

**Table A1:** Sequences of primers for RT-qPCR.

Gene	Forward (5′-3′)	Reverse (5′-3′)
*ACS1*	ACAAGTTCAAGATCACCCAAGC	AGTGCATCCTTTTCTCGTTGAC
*ACO1*	CTGCCCCTCCTGCTCTGT	CTCTGACTGCCTCAAATCTCG
*EXP2*	GCAGAGCAACGCGTACCTCACT	GAGAAGAGGTCGAACAGCGCGA
*EXP8*	TCCTCAAGATCGCCGAGTACC	CAGTTCTGGCCCCAGTTGC
*ERF11*	TGGGTTCAAGGGAATCGGG	TGTTCGTGGGTTCTGTCAAG
*EXP7*	CGTCACCGCCACCAACTTCT	TGGACCCCTTGATCGACACC
*PL1*	CTGGAGGTCGGAAGGGGA	CGGCAGAGACGGTGATGG
*PL2*	AAGACCTGGTTCAGAGGATGCCAA	TGGCTGTTTATAGTGGGAGCAGCA
*PG*	CGGATGAGCAATGTTTCCAACCCA	ACATGGAGAACTGTCGCTGCAAGA
*Actin*	GAGAAGATACAGTGTCTGGA	ATTACCATCGAAATATTAAAAG
